# Tetra­chlorido(1,10-phenanthroline-κ^2^ *N*,*N*′)platinum(IV) monohydrate

**DOI:** 10.1107/S1600536809002694

**Published:** 2009-01-28

**Authors:** Nam-Ho Kim, In-Chul Hwang, Kwang Ha

**Affiliations:** a School of Applied Chemical Engineering, The Research Institute of Catalysis, Chonnam National University, Gwangju 500-757, Republic of Korea; b Department of Chemistry, Pohang University of Science and Technology, Pohang 790-784, Republic of Korea

## Abstract

In the title complex, [PtCl_4_(C_12_H_8_N_2_)]·H_2_O, the Pt^4+^ ion is six-coordinated in a distorted octa­hedral environment by two N atoms of a 1,10-phenanthroline ligand and by four Cl atoms. As a result of the different *trans* effects of the N and Cl atoms, the Pt—Cl bonds *trans* to the N atom are slightly shorter than those *trans* to the Cl atom. The compound displays inter­molecular π–π inter­actions between the six-membered rings, with a centroid–centroid distance of 3.834 Å. There are also weak intra­molecular C—H⋯Cl hydrogen bonds. According to the IR spectrum, solvent water was present in the crystal, but owing to the high thermal motion of the uncoordinated O atom, the H atoms could not be detected.

## Related literature

For details of some other Pt–phenanthroline complexes, see: Buse *et al.* (1977 ▶); Fanizzi *et al.* (1996 ▶). For related Pt–bipyridine complexes, see: Hambley (1986 ▶); Hojjat Kashani *et al.* (2008 ▶).
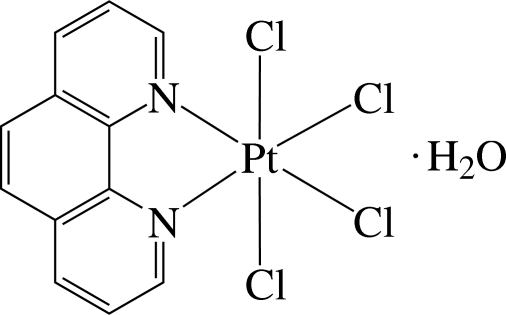


## Experimental

### 

#### Crystal data

[PtCl_4_(C_12_H_8_N_2_)]·H_2_O
*M* *_r_* = 535.11
Orthorhombic, 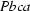

*a* = 14.8481 (19) Å
*b* = 12.4079 (16) Å
*c* = 17.379 (2) Å
*V* = 3201.8 (7) Å^3^
*Z* = 8
Mo *K*α radiation
μ = 9.43 mm^−1^
*T* = 293 (2) K
0.25 × 0.08 × 0.06 mm

#### Data collection

Bruker SMART 1000 CCD diffractometer
Absorption correction: multi-scan (*SADABS*; Bruker, 2000 ▶) *T*_min_ = 0.418, *T*_max_ = 0.568
18465 measured reflections
3521 independent reflections
2414 reflections with *I* > 2σ(*I*)
*R*_int_ = 0.047

#### Refinement

*R*[*F* ^2^ > 2σ(*F* ^2^)] = 0.046
*wR*(*F* ^2^) = 0.141
*S* = 1.02
3521 reflections
181 parameters
H-atom parameters constrained
Δρ_max_ = 1.41 e Å^−3^
Δρ_min_ = −0.56 e Å^−3^

### 

Data collection: *SMART* (Bruker, 2000 ▶); cell refinement: *SAINT* (Bruker, 2000 ▶); data reduction: *SAINT*; program(s) used to solve structure: *SHELXS97* (Sheldrick, 2008 ▶); program(s) used to refine structure: *SHELXL97* (Sheldrick, 2008 ▶); molecular graphics: *ORTEP-3* (Farrugia, 1997 ▶) and *PLATON* (Spek, 2003 ▶); software used to prepare material for publication: *SHELXL97*.

## Supplementary Material

Crystal structure: contains datablocks global, I. DOI: 10.1107/S1600536809002694/fj2191sup1.cif

Structure factors: contains datablocks I. DOI: 10.1107/S1600536809002694/fj2191Isup2.hkl

Additional supplementary materials:  crystallographic information; 3D view; checkCIF report

## Figures and Tables

**Table 1:** Hydrogen-bond geometry (Å, °)

*D*—H⋯*A*	*D*—H	H⋯*A*	*D*⋯*A*	*D*—H⋯*A*
C1—H1⋯Cl2	0.93	2.72	3.298 (10)	121
C10—H10⋯Cl1	0.93	2.74	3.306 (10)	121

**Fig. 1.**
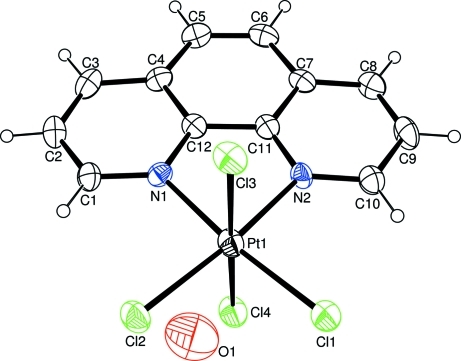
The structure of the title compound, with displacement ellipsoids drawn at the 30% probability level for non-H atoms.

**Fig. 2.**
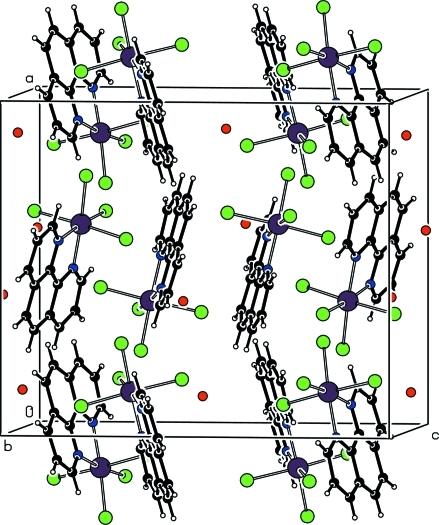
View of the unit-cell contents of the title compound.

## supplementary crystallographic information

### Comment

The asymmetric unit of the title compound, [PtCl_4_(C_12_H_8_N_2_)].H_2_O, contains a neutral Pt^IV^ complex and a water molecule (Fig. 1 and 2). In the complex, the Pt^4+^ ion is six-coordinated in a distorted octahedral environment by two N atoms of the 1,10-phenanthroline ligand and four Cl atoms. The main contribution to the distortion is the tight N1—Pt1—N2 chelate angle (80.1 (2)°), which result in non-linear *trans* axes (<Cl1—Pt1—N1 = 174.0 (2)°, <Cl2—Pt1—N2 = 173.9 (2)° and <Cl3—Pt1—Cl4 = 176.84 (10)°). As a result of the different *trans* effects of the N and Cl atoms, the Pt—Cl bonds *trans* to the N atom (lengths: 2.317 (3) and 2.320 (2) Å) are slightly shorter than bond lengths to mutually *trans* Cl atoms (lengths: 2.343 (3) and 2.335 (3) Å). The compound displays intermolecular π-π interactions between six-membered rings, with a shortest centroid-centroid distance of 3.834 Å and with a dihedral angle between the ring planes of 1.48°. There are also weak intramolecular C—H···Cl hydrogen bonds (Table 1). According to the IR spectrum, water was present in the crystal.

### Experimental

To a solution of K_2_PtCl_6_ (0.3002 g, 0.618 mmol) in H_2_O (20 ml) was added 1,10-phenanthroline (0.1108 g, 0.615 mmol) in MeOH (10 ml), and stirred for 3 h at room temperature. The formed precipitate was separated by filtration and washed with water and MeOH and dried under vacuum, to give a yellow powder (0.1655 g). Crystals suitable for X-ray analysis were obtained by slow evaporation from a CH_2_Cl_2_ solution. IR (KBr): 3424 cm^-1^ (broad).

### Refinement

H atoms were positioned geometrically and allowed to ride on their respective parent atoms [C—H = 0.93 Å and *U*_iso_(H) = 1.2*U*_eq_(C)]. Due to the high thermal motion of the oxygen atom of the solvent H_2_O molecule, the H atoms could neither be located from Fourier difference maps, nor added geometrically.

### Crystal data

**Table:**

[PtCl_4_(C_12_H_8_N_2_)]·H_2_O	*F*(000) = 2000
*M**_r_* = 535.11	*D*_x_ = 2.220 Mg m^−^^3^
Orthorhombic, *P**b**c**a*	Mo *K*α radiation, λ = 0.71073 Å
Hall symbol: -P 2ac 2ab	Cell parameters from 943 reflections
*a* = 14.8481 (19) Å	θ = 3.2–23.2°
*b* = 12.4079 (16) Å	µ = 9.43 mm^−^^1^
*c* = 17.379 (2) Å	*T* = 293 K
*V* = 3201.8 (7) Å^3^	Stick, yellow
*Z* = 8	0.25 × 0.08 × 0.06 mm

### Data collection

**Table:**

Bruker SMART 1000 CCD diffractometer	3521 independent reflections
Radiation source: fine-focus sealed tube	2414 reflections with *I* > 2σ(*I*)
graphite	*R*_int_ = 0.047
φ and ω scans	θ_max_ = 27.1°, θ_min_ = 2.3°
Absorption correction: multi-scan (*SADABS*; Bruker, 2000)	*h* = −18→18
*T*_min_ = 0.418, *T*_max_ = 0.568	*k* = −11→15
18465 measured reflections	*l* = −22→21

### Refinement

**Table:**

Refinement on *F*^2^	Primary atom site location: structure-invariant direct methods
Least-squares matrix: full	Secondary atom site location: difference Fourier map
*R*[*F*^2^ > 2σ(*F*^2^)] = 0.046	Hydrogen site location: inferred from neighbouring sites
*wR*(*F*^2^) = 0.141	H-atom parameters constrained
*S* = 1.02	*w* = 1/[σ^2^(*F*_o_^2^) + (0.0738*P*)^2^ + 11.9979*P*] where *P* = (*F*_o_^2^ + 2*F*_c_^2^)/3
3521 reflections	(Δ/σ)_max_ < 0.001
181 parameters	Δρ_max_ = 1.41 e Å^−^^3^
0 restraints	Δρ_min_ = −0.56 e Å^−^^3^

### Special details

**Table:**

Geometry. All e.s.d.'s (except the e.s.d. in the dihedral angle between two l.s. planes) are estimated using the full covariance matrix. The cell e.s.d.'s are taken into account individually in the estimation of e.s.d.'s in distances, angles and torsion angles; correlations between e.s.d.'s in cell parameters are only used when they are defined by crystal symmetry. An approximate (isotropic) treatment of cell e.s.d.'s is used for estimating e.s.d.'s involving l.s. planes.
Refinement. Refinement of *F*^2^ against ALL reflections. The weighted *R*-factor *wR* and goodness of fit *S* are based on *F*^2^, conventional *R*-factors *R* are based on *F*, with *F* set to zero for negative *F*^2^. The threshold expression of *F*^2^ > σ(*F*^2^) is used only for calculating *R*-factors(gt) *etc*. and is not relevant to the choice of reflections for refinement. *R*-factors based on *F*^2^ are statistically about twice as large as those based on *F*, and *R*- factors based on ALL data will be even larger.

### Fractional atomic coordinates and isotropic or equivalent isotropic displacement parameters (Å^2^)

**Table:**

	*x*	*y*	*z*	*U*_iso_*/*U*_eq_	
Pt1	−0.12471 (3)	0.29079 (3)	0.18568 (2)	0.04495 (16)	
Cl1	−0.27151 (18)	0.3484 (2)	0.20445 (16)	0.0580 (7)	
Cl2	−0.15722 (18)	0.12006 (19)	0.23250 (16)	0.0549 (6)	
Cl3	−0.16047 (19)	0.23631 (19)	0.06024 (15)	0.0537 (6)	
Cl4	−0.0833 (2)	0.3517 (2)	0.30779 (14)	0.0568 (6)	
N1	0.0092 (5)	0.2536 (6)	0.1619 (4)	0.0364 (16)	
N2	−0.0815 (5)	0.4383 (5)	0.1425 (4)	0.0368 (16)	
C1	0.0512 (7)	0.1599 (7)	0.1735 (5)	0.045 (2)	
H1	0.0193	0.1016	0.1932	0.054*	
C2	0.1400 (7)	0.1490 (8)	0.1567 (7)	0.051 (2)	
H2	0.1690	0.0845	0.1679	0.062*	
C3	0.1871 (7)	0.2308 (8)	0.1239 (6)	0.052 (3)	
H3	0.2471	0.2204	0.1105	0.062*	
C4	0.1462 (6)	0.3308 (7)	0.1100 (6)	0.041 (2)	
C5	0.1870 (6)	0.4213 (8)	0.0779 (6)	0.048 (2)	
H5	0.2472	0.4177	0.0633	0.058*	
C6	0.1405 (6)	0.5157 (8)	0.0675 (5)	0.048 (2)	
H6	0.1697	0.5738	0.0448	0.058*	
C7	0.0491 (6)	0.5276 (7)	0.0901 (5)	0.039 (2)	
C8	−0.0011 (6)	0.6212 (7)	0.0837 (6)	0.047 (2)	
H8	0.0249	0.6832	0.0634	0.057*	
C9	−0.0879 (8)	0.6221 (7)	0.1068 (6)	0.056 (3)	
H9	−0.1213	0.6853	0.1028	0.067*	
C10	−0.1284 (6)	0.5293 (7)	0.1368 (6)	0.047 (2)	
H10	−0.1882	0.5311	0.1526	0.057*	
C11	0.0064 (6)	0.4363 (6)	0.1212 (5)	0.0369 (19)	
C12	0.0537 (6)	0.3380 (7)	0.1308 (5)	0.0361 (19)	
O1	0.0973 (14)	0.4296 (19)	0.4629 (12)	0.258 (11)	

### Atomic displacement parameters (Å^2^)

**Table:**

	*U*^11^	*U*^22^	*U*^33^	*U*^12^	*U*^13^	*U*^23^
Pt1	0.0506 (3)	0.0368 (2)	0.0475 (3)	0.00031 (15)	−0.00128 (17)	0.00387 (15)
Cl1	0.0537 (14)	0.0544 (15)	0.0658 (17)	0.0055 (11)	0.0089 (12)	0.0134 (12)
Cl2	0.0589 (14)	0.0404 (12)	0.0653 (17)	−0.0065 (11)	0.0036 (12)	0.0105 (11)
Cl3	0.0648 (15)	0.0489 (13)	0.0475 (15)	−0.0084 (11)	−0.0064 (12)	0.0002 (11)
Cl4	0.0744 (17)	0.0486 (14)	0.0473 (15)	0.0025 (12)	−0.0074 (12)	−0.0021 (11)
N1	0.036 (4)	0.036 (4)	0.037 (4)	−0.001 (3)	0.002 (3)	−0.006 (3)
N2	0.043 (4)	0.024 (3)	0.044 (4)	−0.001 (3)	0.000 (3)	0.004 (3)
C1	0.053 (6)	0.030 (5)	0.053 (6)	0.002 (4)	0.003 (5)	−0.002 (4)
C2	0.056 (6)	0.037 (5)	0.062 (7)	0.008 (4)	−0.008 (5)	−0.001 (5)
C3	0.041 (5)	0.058 (6)	0.056 (6)	0.012 (4)	−0.006 (5)	−0.017 (5)
C4	0.041 (5)	0.042 (5)	0.040 (5)	−0.007 (4)	0.002 (4)	−0.011 (4)
C5	0.040 (5)	0.056 (6)	0.048 (6)	−0.010 (4)	0.003 (4)	−0.008 (5)
C6	0.058 (6)	0.050 (6)	0.037 (5)	−0.017 (4)	0.001 (4)	0.001 (4)
C7	0.054 (5)	0.036 (5)	0.027 (4)	−0.010 (4)	−0.006 (4)	−0.004 (3)
C8	0.062 (6)	0.032 (5)	0.048 (6)	−0.013 (4)	−0.002 (5)	−0.003 (4)
C9	0.084 (7)	0.025 (4)	0.059 (7)	0.003 (5)	−0.010 (6)	0.000 (4)
C10	0.053 (5)	0.039 (5)	0.050 (6)	0.002 (4)	−0.002 (5)	0.002 (4)
C11	0.048 (5)	0.032 (4)	0.030 (5)	−0.002 (4)	−0.007 (4)	−0.006 (4)
C12	0.044 (5)	0.032 (4)	0.032 (5)	−0.006 (4)	−0.006 (4)	−0.005 (3)
O1	0.33 (3)	0.29 (3)	0.151 (17)	0.06 (2)	0.028 (17)	0.043 (17)

### Geometric parameters (Å, °)

**Table:**

Pt1—N2	2.080 (7)	C3—H3	0.9300
Pt1—N1	2.083 (7)	C4—C5	1.393 (13)
Pt1—Cl1	2.317 (3)	C4—C12	1.424 (12)
Pt1—Cl2	2.320 (2)	C5—C6	1.372 (13)
Pt1—Cl4	2.335 (3)	C5—H5	0.9300
Pt1—Cl3	2.343 (3)	C6—C7	1.421 (13)
N1—C1	1.335 (11)	C6—H6	0.9300
N1—C12	1.351 (11)	C7—C8	1.384 (13)
N2—C10	1.330 (11)	C7—C11	1.405 (11)
N2—C11	1.357 (11)	C8—C9	1.351 (14)
C1—C2	1.357 (13)	C8—H8	0.9300
C1—H1	0.9300	C9—C10	1.400 (14)
C2—C3	1.358 (14)	C9—H9	0.9300
C2—H2	0.9300	C10—H10	0.9300
C3—C4	1.402 (13)	C11—C12	1.417 (12)
			
N2—Pt1—N1	80.1 (3)	C4—C3—H3	119.7
N2—Pt1—Cl1	94.0 (2)	C5—C4—C3	126.5 (8)
N1—Pt1—Cl1	174.0 (2)	C5—C4—C12	118.0 (8)
N2—Pt1—Cl2	173.9 (2)	C3—C4—C12	115.5 (8)
N1—Pt1—Cl2	93.8 (2)	C6—C5—C4	121.5 (9)
Cl1—Pt1—Cl2	92.10 (9)	C6—C5—H5	119.2
N2—Pt1—Cl4	87.8 (2)	C4—C5—H5	119.2
N1—Pt1—Cl4	90.0 (2)	C5—C6—C7	122.2 (8)
Cl1—Pt1—Cl4	91.14 (10)	C5—C6—H6	118.9
Cl2—Pt1—Cl4	91.81 (10)	C7—C6—H6	118.9
N2—Pt1—Cl3	89.3 (2)	C8—C7—C11	117.7 (8)
N1—Pt1—Cl3	88.2 (2)	C8—C7—C6	125.4 (8)
Cl1—Pt1—Cl3	90.39 (10)	C11—C7—C6	117.0 (8)
Cl2—Pt1—Cl3	90.90 (9)	C9—C8—C7	119.8 (9)
Cl4—Pt1—Cl3	176.84 (10)	C9—C8—H8	120.1
C1—N1—C12	120.5 (8)	C7—C8—H8	120.1
C1—N1—Pt1	127.5 (6)	C8—C9—C10	120.9 (9)
C12—N1—Pt1	112.0 (6)	C8—C9—H9	119.5
C10—N2—C11	120.0 (7)	C10—C9—H9	119.5
C10—N2—Pt1	127.7 (6)	N2—C10—C9	120.0 (9)
C11—N2—Pt1	112.3 (5)	N2—C10—H10	120.0
N1—C1—C2	120.5 (9)	C9—C10—H10	120.0
N1—C1—H1	119.7	N2—C11—C7	121.6 (8)
C2—C1—H1	119.7	N2—C11—C12	117.4 (7)
C1—C2—C3	121.1 (9)	C7—C11—C12	121.0 (8)
C1—C2—H2	119.5	N1—C12—C11	118.2 (8)
C3—C2—H2	119.5	N1—C12—C4	121.6 (8)
C2—C3—C4	120.7 (9)	C11—C12—C4	120.2 (8)
C2—C3—H3	119.7		
			
N2—Pt1—N1—C1	179.4 (8)	C11—C7—C8—C9	−0.1 (13)
Cl2—Pt1—N1—C1	−0.2 (8)	C6—C7—C8—C9	179.5 (9)
Cl4—Pt1—N1—C1	91.6 (7)	C7—C8—C9—C10	−0.6 (15)
Cl3—Pt1—N1—C1	−91.0 (7)	C11—N2—C10—C9	1.7 (14)
N2—Pt1—N1—C12	−1.2 (6)	Pt1—N2—C10—C9	179.3 (7)
Cl2—Pt1—N1—C12	179.2 (5)	C8—C9—C10—N2	−0.2 (15)
Cl4—Pt1—N1—C12	−89.0 (5)	C10—N2—C11—C7	−2.5 (13)
Cl3—Pt1—N1—C12	88.4 (5)	Pt1—N2—C11—C7	179.6 (6)
N1—Pt1—N2—C10	−177.2 (8)	C10—N2—C11—C12	178.2 (8)
Cl1—Pt1—N2—C10	4.2 (8)	Pt1—N2—C11—C12	0.3 (9)
Cl4—Pt1—N2—C10	−86.8 (8)	C8—C7—C11—N2	1.7 (12)
Cl3—Pt1—N2—C10	94.6 (8)	C6—C7—C11—N2	−178.0 (8)
N1—Pt1—N2—C11	0.5 (6)	C8—C7—C11—C12	−179.0 (8)
Cl1—Pt1—N2—C11	−178.1 (6)	C6—C7—C11—C12	1.3 (12)
Cl4—Pt1—N2—C11	90.9 (6)	C1—N1—C12—C11	−178.7 (8)
Cl3—Pt1—N2—C11	−87.7 (6)	Pt1—N1—C12—C11	1.8 (9)
C12—N1—C1—C2	2.3 (13)	C1—N1—C12—C4	−0.6 (12)
Pt1—N1—C1—C2	−178.3 (7)	Pt1—N1—C12—C4	180.0 (6)
N1—C1—C2—C3	−3.7 (16)	N2—C11—C12—N1	−1.4 (12)
C1—C2—C3—C4	3.2 (16)	C7—C11—C12—N1	179.2 (7)
C2—C3—C4—C5	179.0 (10)	N2—C11—C12—C4	−179.6 (8)
C2—C3—C4—C12	−1.4 (14)	C7—C11—C12—C4	1.0 (12)
C3—C4—C5—C6	−179.5 (9)	C5—C4—C12—N1	179.7 (8)
C12—C4—C5—C6	1.0 (14)	C3—C4—C12—N1	0.1 (12)
C4—C5—C6—C7	1.4 (15)	C5—C4—C12—C11	−2.2 (13)
C5—C6—C7—C8	177.8 (9)	C3—C4—C12—C11	178.2 (8)
C5—C6—C7—C11	−2.6 (13)		

### Hydrogen-bond geometry (Å, °)

**Table:**

*D*—H···*A*	*D*—H	H···*A*	*D*···*A*	*D*—H···*A*
C1—H1···Cl2	0.93	2.72	3.298 (10)	121
C10—H10···Cl1	0.93	2.74	3.306 (10)	121
